# General Intermediates for the Synthesis of 6-C-Alkylated DMDP-Related Natural Products

**DOI:** 10.3390/molecules18066723

**Published:** 2013-06-06

**Authors:** Mu-Hua Huang, Yi-Xian Li, Yue-Mei Jia, Chu-Yi Yu

**Affiliations:** 1 Beijing National Laboratory for Molecular Science (BNLMS), CAS Key Laboratory of Molecular Recognition and Function, Institute of Chemistry, Chinese Academy of Sciences, Beijing 100191, China; 2 School of Materials Science and Engineering, Beijing Institute of Technology, Beijing 100081, China

**Keywords:** homo-DMDP, broussonetine G, homoDMDP-7-O-apioside, glycosidase inhibitors, cyclic nitrones

## Abstract

Protected L-homoDMDP *en-***8** and its C-6 epimer *en*-**7** were prepared through two different pathways starting from the vinylpyrrolidine *en*-**9**. Based on the NMR and X-ray analysis, the stereochemistry of homoDMDP at C-6 was confirmed to be consistent with reported data. Compounds *en-***7** and *en*-**8** are general intermediates for the synthesis of a series of 6-C-alkylated DMDP-related natural products, such as broussonetine G, homoDMDP-7-*O*-apioside, homoDMDP-7-*O*-β-D-xyloside and so on.

## 1. Introduction

**Figure 1:**
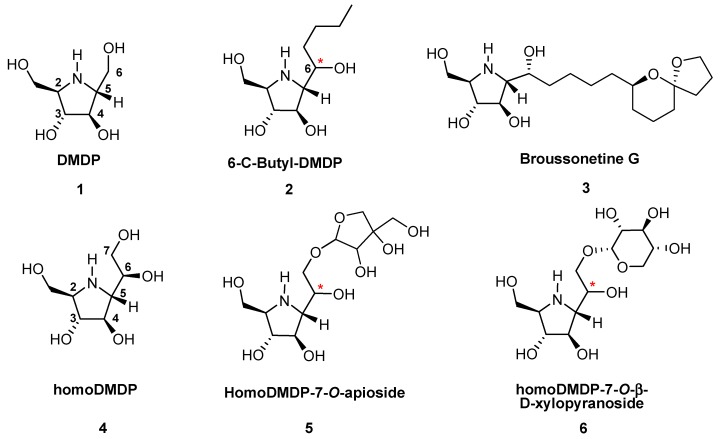
DMDP, homoDMDP and 6-*C*-alkyl-DMDP natural products.

The naturally occurring polyhydroxylated alkaloid (*2R*,*3R*,*4R*,*5R*)-2,5-dihydroxymethyl-3,4-dihydroxypyrrolidine (DMDP, **1**, Figure 1) [1] has become a key iminosugar in the field of glycosidase inhibitors [2,3,4]. 6*-C-*Butyl-DMDP (**2**, Figure 1) [5] and broussonetine G (**3**, Figure 1) [6,7,8] represent a class of 6*-C-*alkyl-DMDP natural products with high β-glycosidase inhibitory properties. Another 6*-C-*alkyl-DMDP example is 2,5-dideoxy-2,5-imino-*glycero*-D,L-*manno*-heptitol (homo-DMDP, **4**, Figure 1), which was synthesized by the Wong group [9] in 1995 before it was isolated in 1997 [10] from Hyacinthaceae plants. In addition, 7-*O*-glycosylated homoDMDP natural products such as homoDMDP-7-*O*-apioside (**5**, Figure 1) and homoDMDP-7-*O*-β-D-xyloside (**6**, Figure 1), isolated from bluebell, also showed very highly selective glycosidase inhibition properties [11]. Interestingly, the stereochemistry at C-6 of the above mentioned 6-*C*-alkyl-DMDP natural products was not reported upon their isolation owing to limitations on NMR analysis due to the scarce amounts of substance available from natural sources. An unambiguous stereochemistry elucidation of the homoDMDP-related natural products is needed for establishing the structure-activity relationships of these compounds in medicinal chemistry, and a collective synthesis of these natural products based on a common intermediate would be of great significance [12] for speeding up the chemical biology study of these fascinating molecules.

In recent years, some of the synthetic L-enantiomers of iminosugars, were found to be even more powerful and specific glycosidase inhibitors than the natural products, e.g., L-DMDP* vs.* DMDP [13], (+)-steviamine* vs.* natural (-)-steviamine [14]. These experimental results provided a promising new direction for pharmaceutical chemistry. However, a substantive explanation for this fact requires more information about the stereochemistry of the relevant compounds. In order to promote research on iminosugars, our group has developed a powerful strategy for the synthesis of DMDP-related natural products or analogues via cyclic nitrone intermediates [15], which has led to easy access to a series of DMDP-related iminosugars [13,14,16,17,18,19,20,21], as well as glycosidase inhibitors.

Although Wong *et al.* had indicated the absolute configuration of homoDMDP to be (*2R*,*3R*,*4R*,*5R*,*6S*) based on their enantiospecific synthesis of a series of five-membered iminosugars [22], no direct experimental proof was available until our group reported the synthesis of L-homoDMDP [19]. There, we disclosed the stereochemistry of L-homoDMDP determined by analyzing the 2D-NMR of an oxazinone intermediate, a conformation-locked L-homoDMDP. In this article, we report a further confirmation of L-homoDMDP’s stereochemistry based on X-ray and 2D-NMR analysis of its oxazolidinone derivatives. In addition, both epimers of protected L-homoDMDP at C-6 were obtained readily, which are common intermediates for enantiomers of 6-*C*-alkyl-DMDP natural products such as homoDMDP-7-*O*-apioside and homoDMDP-7-*O*-β-D-xyloside.

## 2. Results and Discussion

6*-C-*Alkyl-DMDP iminosugars have attracted lots of attention, and several syntheses are available in the literature [7,22]. However, these syntheses are either tedious or the method was only suitable for a specific compound. In order to solve the material supply problem and speed up the study of glycosidase inhibition, a practical synthesis of this family of natural products is in great demand. In this regard, the collective synthesis strategy [12] relying on an advanced common intermediate provides a good opportunity. In addition, a full assignment of the stereochemistry of 6*-C-*alkyl-DMDP iminosugars requires both epimers.

**Scheme 1:**
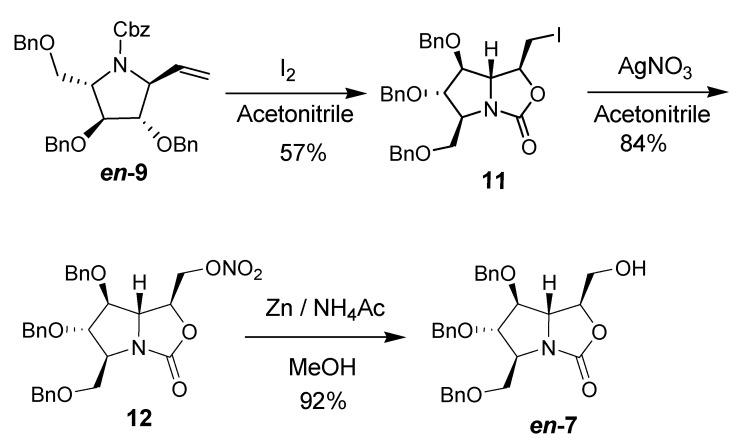
Synthesis of *en-***7**.

Taking homo-DMDP-7-*O*-apioside and homo-DMDP-7-*O*-β-D-xylopyranoside as examples, we envisioned that it is necessary to obtain both C-6 epimers **7** and **8**, which could be reached from the same intermediate **9** (Scheme 1). Furthermore, conformation-locked molecules **7** and **8** are very good intermediates for stereochemistry assignment based on 2D-NMR analysis, and all the isomers or intermediates represent interesting medicinal chemistry targets. From a retrosynthesis point of view, homoDMDP-related natural products **5** and **6** could be synthesized via glycosylation of alcohol **7** or **8**, which could be accessed from all-*trans* substituted pyrrolidine **9 **by alkene oxidation. Based on the well-developed chemistry of highly stereoselective addition of organometallic reagents to cyclic nitrones [23,24,25], pyrrolidine **9** could be prepared on a large scale with extreme purity.

**Figure 2:**
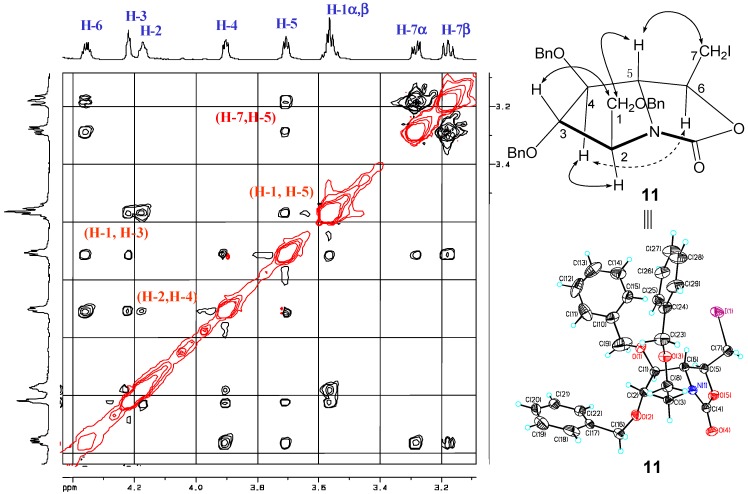
Stereochemistry elucidation of **11** by NOESY and X-ray analysis.

With grams of vinyl-containing pyrrolidine *en-***9** [19] in hand, the synthesis of *en*-**7** and ***en*-8** was attempted (Scheme 1). The terminal alkene *en-***9** was treated with iodine in acetonitrile, and the oxazolidinone **11** was obtained in 57% yield, with structure supported by its IR data (ν_max_ 1,762 cm^−1^) and ^13^C-NMR spectrum (δ 159.98 ppm). Its relative configuration was studied by its NOESY spectrum based on the full assignment of all protons through ^1^H-NMR and 2D-NMR (COSY, HMBC). The strong correlations observed between H-3/H-1 as well as H-5/H-1 revealed that H-1,H-3 and H-5 were on the underside of the pyrrolidine ring (Figure 2), while the strong correlations between H-2/H-4 showed they were on the same side.* i.e.*, the pyrrolidine was all-*trans* substituted, which was consistent with the previous results reported by our group. The correlations between H-5/H-1 as well as H-5/H-7 showed the C(7)H_2_I was on the upper side of the oxazolidinone ring. Furthermore, the above determination of relative as well as absolute configuration of the oxazolidinone **11** was unambiguously confirmed to be (*2S*,*3S*,*4S*,*5R*,*6S*) by X-ray diffraction [26]. The treatment of iodide **11** by silver nitrate followed by reductive hydrolysis [27] furnished the alcohol *en-***7** in a total yield of 77% over two steps. Since during these two steps the stereocenters have not been touched, the streochemistry of *en-***7** was determined to be (*2S*,*3S*,*4S*,*5S*,*6S*).

**Scheme 2:**
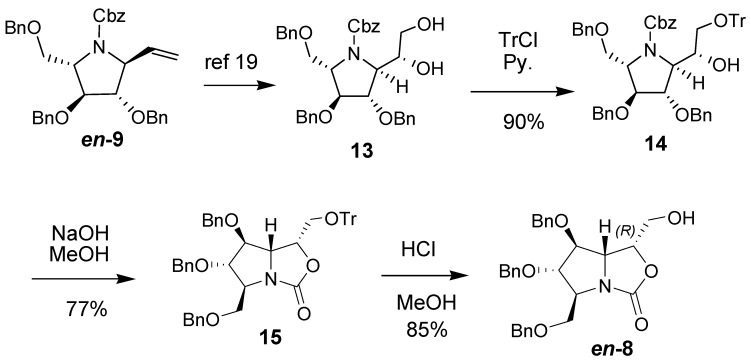
Synthesis of *en-***8**.

In the course of the synthesis of L-homoDMDP, we were delighted to find that the major product **13** (Scheme 2) obtained from dihydroxylation on terminal alkene *en-***9** was the right diastereomer for L-homoDMDP, which was prepared by the hydrogenation on diol **13** to remove the protecting groups. The stereochemistry of L-homoDMDP was confirmed to be (*2S*,*3S*,*4S*,*5S*,*6R*) based on a conformation-locked oxazinone intermediate [19]. In order to provide more evidence for the stereochemistry confirmation of homoDMDP, as well as common intermediates in the collective synthesis of homoDMDP-related pyrrolidine natural products, we decided to make the correseponding oxazolidinone derivatives. The diol **13** was selectively protected as trityl ether **14**, the secondary alcohol **14** was then treated with alkaline solution, and the oxazolidinone **15** was obtained in 69% yield over two steps. After treatment of trityl ether **15 **with mineral acid, the alcohol *en-***8** was obtained in 85% yield (Scheme 2). Conformation of the oxazolidinone structure was supported by its IR data (ν_max_ 1,758 cm^−1^) and ^13^C-NMR spectrum (δ 160.03 ppm).

**Figure 3:**
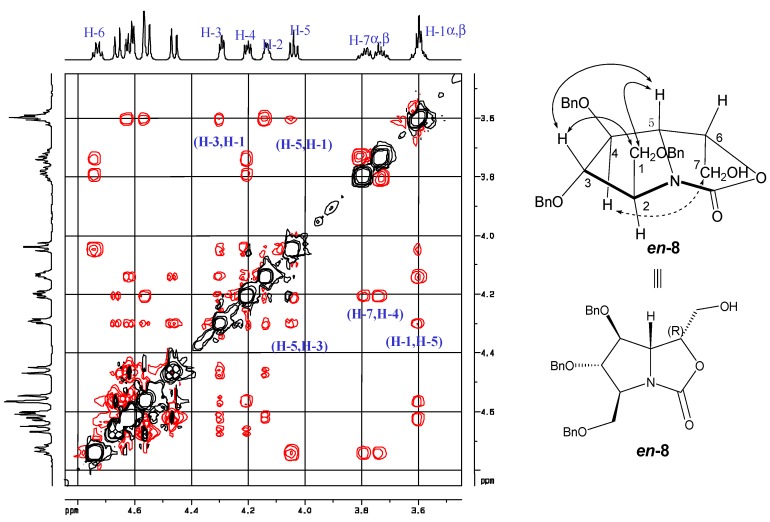
Stereochemistry elucidation of *en-***8** by NOESY.

Similarly, the relative configuration of alcohol *en-***8** was assigned unambiguously through NOESY spectrum (Figure 3). The strong correlations observed between H-3/H-1, H-5/H-1, as well as H-5/H-3 revealed H-1, H-3 and H-5 were on the upper side of the pyrrolidine ring,* i.e.*, the pyrrolidine was all-*trans*-substituted. The correlations between H-7/H-4 showed the C(7)H_2_OH was on the underside of the oxazolidinone ring. Based on the above analysis as well as comparison with *en-***7**, the stereochemistry of *en-***8** was deduced to be (*2S*,*3S*,*4S*,*5S*,*6R*), and the configuration of homoDMDP was determined as (*2R*,*3R*,*4R*,*5R*,*6S*), which was consistent with reported data [19].

With both C-6 epimers *en-***7** and *en-***8** in hand, the syntheses of the enantiomers of homo-DMDP-7-O-apioside and homo-DMDP-7-O-β-D-xylopyranoside by glycosylation of *en-***7** and *en-***8** were on the right course. In addition, iodide **11** represents an ideal intermediate for the total synthesis of enantiomers of 6*-C-*alkyl-DMDP natural products such as L-6*-C-*butyl-DMDP and (+)-broussonetine G. For the total synthesis of the natural 6*-C-*alkyl-DMDP pyrrolidines, we are currently working on the synthesis of cyclic nitrone **10**. The relevant results will be reported in due course.

## 3. Experimental

### 3.1. General

All reagents were used as received from commercial sources without further purification or prepared as described in the literature. Tetrahydrofuran was distilled from sodium and benzophenone immediately before use. Reaction mixtures were stirred using Teflon-coated magnetic stir bars. Analytical TLC was performed with 0.20 mm silica gel 60F plates with 254 nm fluorescent indicator. Plates were visualized by ultraviolet light and treatment with Pancaldi reagent [(NH_4_)_6_MoO_4_, Ce(SO_4_)_2_, H_2_SO_4_, H_2_O] or 0.5% ninhydrin in acetone stain, followed by gentle heating. Chromatographic purification of products was carried out by flash chromatography on silica gel (230–400 mesh) as well as on the 001 × 7 (732^#^) strong acid styrene cation exchange resin (Tianjin Kermel Chemical Reagent Development Center, Tianjin, China). Melting points were determined using an Electrothermal melting point apparatus. Both melting points and boiling points are uncorrected. Infrared spectra were recorded on a JASCO FT/IR-480 plus Fourier transform spectrometer. NMR spectra were measured in CDCl_3_ (with TMS as internal standard) or D_2_O on a Bruker AV300 and AV600 magnetic resonance spectrometers. Chemical shifts (δ) are reported in ppm, and coupling constants (*J*) are in Hz. The following abbreviations were used to explain the multiplicities: s = singlet, d = doublet, t = triplet, q = quartet, m = multiplet. High-resolution mass spectra (HRMS) were recorded on an APEXII FT-ICR (SIMS) mass spectrometer. Polarimetry was carried out using an Optical Activity AA-10R polarimeter and the measurements were made at the sodium D-line with a 0.5 dm pathlength cell. Concentrations (c) are given in gram per 100 mL. Single-crystal X-ray diffraction was collected on a Rigaku RAPID-AUTO diffractometer.

*(1S*,*5S*,*6S*,*7S*,*7aR)-6*,*7-bis(Benzyloxy)-5-((benzyloxy)methyl)-tetrahydro-1-(iodomethyl)pyrrolo[1*,*2-c]oxazol-3(1H)-one *(**11**). To a solution of alkene *en-***9** (0.22 g, 0.39 mmol) in acetonitrile (3 mL), iodine (0.36 g, 1.4 mmol) was slowly added, and the resulting purple mixture was stirred at r.t. for 3.5 h. The reaction was then quenched by adding sat. aqueous Na_2_S_2_O_3_, and extracted with EtOAc. The combined organic extracts were dried over anhydrous Na_2_SO_4_, concentrated, the residue was purified by FCC (silica gel, eluted with 10%–20% EtOAc in petroleum ether) to give the title oxazolidinone **11** as a colorless crystalline product (0.13 g, yield 57%). IR(KBr, cm^−1^)1,767 (vs), 1,455 (w), 1,362 (w), 1,213 (w), 1,100 (w). [α]^25^*_D_* = +55.8° (*c* 0.86, CHCl_3_). ^1^H-NMR (CDCl_3_, 600 MHz): δ (ppm) 7.35–7.25 (m, 15H), 4.64–4.44 (m, 6H), 4.37–4.34 (m, 1H), 4.22 (t, 1H, *J *= 2.7 Hz), 4.17 (t, 1H, *J *= 6.6 Hz), 3.90 (dd, 1H, *J *= 3.8 and 5.5 Hz), 3.71 (t, 1H, *J *= 4.3 Hz), 3.59–3.53 (m, 2H), 3.28 (dd, 1H, *J *= 4.3 and 10.4 Hz), 3.18 (dd, 1H, *J *= 8.3 and 10.1 Hz). ^13^C-NMR (CDCl_3_, 75 MHz): δ (ppm) 160.00, 137.84, 137.36, 137.30, 128.64, 128.51, 128.45, 128.21, 127.96, 127.91, 127.78, 127.70, 87.55, 84.99, 78.26, 73.30, 72.74, 71.94, 69.32, 68.12, 62.76, 5.90. DEPT-135 (CDCl_3_, 75 MHz) δ (ppm): Positive: 128.64, 128.51, 128.45, 128.22, 127.96, 127.91, 127.78, 127.70, 87.55, 84.98, 78.26, 68.11, 62.75; Negative: 73.30, 72.74, 71.94, 69.32, 5.91. FTICR-MS *m/z*: 600.1243 [M+H]^+^ (C_29_H_31_NO_5_I^+^ requires 600.1241).

*(1S*,*5S*,*6S*,*7S*,*7aR)-6*,*7-bis(Benzyloxy)-5-((benzyloxy)methyl)-tetrahydro-1-(O-nitroate-methyl)pyrrolo[1*,*2-c]oxazol-3(1H)-one *(**12**). To a solution of iodide **11** (0.21 g, 0.35 mmol) in acetonitrile (6 mL) silver nitrate (0.18 g, 1.0 mmol) was added, the resulting reaction mixture was heated to reflux for 40 h. The resulting solid was filtered off, the filtrates was concentrated, the residue was purified by FCC (silica gel, eluted with 10-30% EtOAc in petroleum ether) to give the nitrate **12** (0.16 g, 84% yield) as colorless syrup. IR (KBr, cm^−1^)：2,864 (w), 1,765 (vs), 1,638 (s), 1,279 (s), 1,096 (m), 739 (m), 698 (m). [α]^25^*_D_* = +22.1° (*c* 1.0, CHCl_3_). ^1^H-NMR (CDCl_3_, 300 MHz): δ (ppm) 7.36–7.19 (m, 15H), 4.69–4.36 (m, 9H), 4.28 (t, 1H, *J *= 3.3 Hz), 4.15 (d, 1H, *J *= 2.9 Hz), 3.90 (dd, 1H, *J* = 4.0 and 6.0 Hz), 3.68 (d, 1H, *J *= 6.1 Hz), 3.58 (d, 2H, *J *= 5.4 Hz). ^13^C-NMR (CDCl_3_, 75 MHz): δ (ppm) 159.30, 137.75, 137.24, 128.78, 128.55, 128.48, 128.43, 128.07, 127.98, 127.93, 127.85, 127.71, 87.66, 85.30, 74.76, 73.35, 72.80, 72.18, 71.88, 69.43, 64.31, 62.69. DEPT-135 (CDCl_3_, 75 MHz) δ (ppm): Positive: 128.78, 128.55, 128.48, 128.06, 127.99, 127.93, 127.85, 127.71, 87.65, 85.30, 74.76, 64.30, 62.69; Negative: 73.35, 72.80, 72.18, 71.88, 69.42. FTICR-MS *m/z*: 535.2079 [M+H]^+^ (C_29_H_31_N_2_O_8_^+^ requires 535.2075).

*(1S*,*5S*,*6S*,*7S*,*7aS)-6*,*7-bis(Benzyloxy)-5-((benzyloxy)methyl)-tetrahydro-1-(hydroxymethyl)pyrrolo[1*,*2-c]oxazol-3(1H)-one *(*en-***7**). To a suspension of nitrate **12** (0.10 g, 0.19 mmol) and ammonium acetate (0.08 g, 1 mmol) in MeOH (5 mL) was added zinc powder (0.11 g, 1 mmol). The resulting mixture was stirred at r. t. The solvents were removed *in vacuo*, the residue was purified via FCC (silica gel, eluted with 10%–40% EtOAc in petroleum ether) to give the alcohol *en-***7** (87 mg, yield 92%) as a colorless syrup. IR (KBr, cm^−^^1^): 3,443 (w), 3,062 (vw), 3,031 (w), 2,928 (w), 2,867 (w), 1,758 (vs), 1,101 (m), 1,070 (m). [α]^25^*_D_* = +14.2° (*c* 1.1, CHCl_3_). ^1^H-NMR (CDCl_3_, 300 MHz): δ (ppm) 7.31–7.14 (m, 15H), 4.31 (dd, 1H, *J *= 4.0 and 4.5 Hz), 4.22 (t, 1H, *J *= 3.0 Hz), 4.18–4.09 (m, 1H), 3.88 (dd, 1H, *J *= 3.9 and 5.9 Hz), 3.76–3.66 (m, 2H), 3.63–3.49 (m, 3H), 1.96 (t, 1H, *J *= 6.5 Hz, OH). ^13^C-NMR (CDCl_3_, 75 MHz): δ (ppm) 160.24, 137.85, 137.43, 137.32, 128.65, 128.49, 128.44, 128.20, 127.94, 127.80, 127.77, 127.70, 87.89, 85.51, 80.05, 73.28, 72.50, 72.01, 69.40, 63.96, 63.55, 62.46. DEPT-135 (CDCl_3_, 75 MHz) δ (ppm) Positive: 128.65, 128.49, 128.44, 128.21, 127.95, 127.80, 127.77, 127.70, 87.88, 85.50, 80.06, 63.95, 62.46; Negative: 73.27, 72.50, 72.01, 69.39, 63.54. FTICR-MS *m/z*: 490.2228 [M+H]^+^ (C_29_H_32_NO_6_+ requires 490.2224).

*(2S*,*3S*,*4S*,*5S)-Benzyl 3*,*4-bis(benzyloxy)-2-((benzyloxy) methyl)-5-((R)-1*,*2-dihydroxyethyl)pyrrolidine-1-carboxylate *(**14**). To a solution of diol (0.22 g, 0.4 mmol) in DCM (3 mL) was added pyrridine (1 mL) and trityl chloride (0.22 g, 0.8 mmol). The resulting reaction mixture was stirred at r.t. for 14 h. The solvents were removed *in vacuo*, the residue was dissolved in EtOAc and HCl (1 N). After extraction, the combined organics were dried over anhydrous Na_2_SO_4_, concentrated, the residue was purified by FCC (silica gel, elutedwith 10%–30% EtOAc in petroleum ether) to give the trityl ether **14** (0.28 g, yield 90%). IR (KBr, cm^−1^):3,435 (s), 1,702 (vs), 1,075 (vs). [α]^25^*_D_* = +64.8° (*c* 1.3, CHCl_3_). ^1^H-NMR (CDCl_3_, 300 MHz): δ (ppm) 7.56–7.03 (m, 35H), 5.36–5.14 (m, 2H), 4.80 (brs, 0.5 H), 4.69–4.07 (m, 11H), 3.90 (brs, 0.5 H), 3.85 (dd, 1H, *J *= 4.1 and 8.9 Hz), 3.63–3.54 (m, 1H), 3.51–3.41 (m, 1H), 3.06 (t, 0.5H, *J *= 8.8 Hz), 2.92 (t, 0.5H, *J *= 8.9 Hz). ^13^C-NMR (CDCl_3_, 75 MHz): δ (ppm) 154.67, 154.50, 143.83, 143.70, 138.42, 138.16, 137.55, 137.41, 136.44, 136.24, 128.80, 128.66, 128.61, 128.39, 128.23, 128.00, 127.89, 127.68, 127.62, 127.57, 127.35, 127.26, 127.08, 127.05, 86.99, 86.90, 82.09, 81.62, 81.41, 80.68, 73.16, 73.09, 71.51, 71.42, 68.77, 68.60, 67.52, 67.45, 67.34, 67.17, 66.70, 65.97, 65.74, 63.35, 63.06. DEPT-135 (CDCl_3_, 300 MHz): δ (ppm) positive 128.79, 128.66, 128.61, 128.39, 128.33, 128.18, 128.13, 128.00, 127.89, 127.61, 127.57, 127.35, 127.26, 127.08, 82.09, 81.62, 81.41, 80.68, 68.77, 67.52, 67.30, 66.70, 63.35, 63.05. negative 73.16, 73.09, 71.51, 71.42, 68.60, 67.44, 67.34, 67.16, 65.97, 65.74. FTICR-MS *m/z*: 840.3904 [M+H]^+^ (C_55_H_54_NO_7_^+^ requires 862.3895); 862.3728 [M+Na]^+^ (C_55_H_53_NNaO_7_^+^ requires 862.3714).

*(1R*,*5S*,*6S*,*7S*,*7aS)-6*,*7-bis(Benzyloxy)-5-((benzyloxy)methyl)-tetrahydro-1(hydroxymethyl)pyrrolo[1*,*2-c]-oxazol-3(1H)-one *(*en-***8**). To a solution of alcohol **14** (64 mg, 0.0076 mmol) in MeOH (3 mL) was added KOH (80 mg, 1.5 mmol).The resulting reaction mixture was stirred at r.t. for 10 min, and then quenched by adding HCl (1 N, 5 mL). The mixture was extracted with EtOAc, the combined organics were dried over anhydrous Na_2_SO_4_, concentrated, and the residue was purified by FCC (silica gel, eluted with 10% EtOAc in petroleum ether) to give the oxazolidinone **15** (43 mg, yield 77%) as a colorless oil. IR (KBr, cm^−1^): 3,061 (w), 3,031 (w), 1,762 (vs), 1,494 (w), 1,450 (w), 1,364 (w), 1,211 (w), 1,093 (m). [α]^25^*_D_* = +2.9° (*c* 0.69 , CHCl_3_). ^1^H-NMR (CDCl_3_, 600 MHz): δ (ppm) 7.37 (d, 6H, ArH), 7.26–7.13 (m, 20H, ArH), 7.09 (d, 2H, *J *= 5.2 Hz, ArH), 6.91 (d, 2H, *J *= 6.9 Hz, ArH), 4.74 (dd, 1H, *J *= 6.2 and 12.7 Hz, H-1), 4.46 (ABQ, 2H, *J *= 12.0 Hz, PhCH_2_O), 4.37 (d, 1H, *J *= 11.8 Hz, PhCH_2_O), 4.23 (d, 1H, *J *= 11.8 Hz, PhCH_2_O), 4.24 (d, 1H, *J *= 11.2 Hz, PhCH_2_O), 4.06 (d, 1H, *J *= 11.1 Hz, PhCH_2_O), 4.08-4.05 (m, 2H, H-5 and H-6), 3.93 (t, 1H, *J *= 7.2 Hz, H-7a), 3.87 (dd, 1H, *J *= 3.6 and 6.4 Hz, H-7), 3.50 (d, 2H, *J *= 5.7 Hz H-9), 3.38 (dd, 1H, *J *= 6.3 and 10.5 Hz, H-8), 3.28 (dd, 1H, *J *= 4.4 and 10.5 Hz, H-8). ^13^C-NMR (CDCl_3_, 75 MHz): δ (ppm) 160.07, 143.27, 137.81, 137.41, 137.30, 137.41, 137.30, 128.64, 128.41, 128.33, 127.97, 127.80, 127.75, 127.67, 127.46, 127.24, 87.31, 85.84, 83.19, 77.44, 76.59, 75.29, 73.28, 72.24, 71.66, 69.63, 64.09, 62.67, 62.37. FTICR-MS *m/z*: 732.3309 [M+H]^+^ (C_48_H_46_NO_6_^+^ requires 732.3319).

*(1R*,*5S*,*6S*,*7S*,*7aS)-6*,*7-bis(Benzyloxy)-5-((benzyloxy)methyl)-tetrahydro-1(hydroxymethyl)pyrrolo[1*,*2-c]-oxazol-3(1H)-one* (*en-***8**). To a solution of oxazolidinone **15** (102 mg, 0.014 mmol) in MeOH (5 mL) was added 5 drops of concentrated HCl, the reaction mixture was stirred at r.t. for 10 mins. TLC revealed the complete disappearance of starting material, the reaction was quenched by adding sat. aqueous NaHCO_3_. The mixture was extracted with EtOAc, the combined organics were dried over anhydrous Na_2_SO_4_, concentrated, the residue was purified by FCC (silica gel, eluted with 10%–50% EtOAc in petroleum ether) to give the oxazolidinone *en-***8** (58 mg, yield 85%) as a colorless syrup. IR (KBr, cm^−1^) 3,442 (w), 3,062 (vw), 3,031 (vw), 2,868 (w), 1,758 (vs), 1,497 (vw), 1,454 (w), 1,364 (w), 1,209 (w), 1,071 (m). [α]^25^*_D_* = –5.1° (*c *1.9, CHCl_3_); ^1^H-NMR (CDCl_3_, 600 MHz): δ (ppm) 7.35–7.24 (m, 15H), 4.71 (dd, 1H, *J *= 5.9 and 13.6 Hz), 4.63 (d, 1H, *J *= 11.4 Hz), 4.59 (d, 1H, *J *= 12.0 Hz), 4.59 (d, 1H, *J *= 11.5 Hz), 4.53 (d, 2H, *J *= 12.4 Hz), 4.43 (d, 1H, *J *= 11.6 Hz), 4.27 (dd, 1H, *J *= 3.8 and 4.5 Hz), 4.18 (dd, 1H, *J *= 4.8 and 8.0 Hz), 4.11 (dd, 1H, *J *= 5.0 and 8.7 Hz), 4.02 (t, 1H, *J *= 7.9 Hz), 3.78–3.70 (m, 2H), 3.60–3.55 (m, 2H), 2.23 (t, 1H, *J *= 6.6 Hz, OH). ^13^C-NMR (CDCl_3_, 75 MHz): δ (ppm) 160.03, 137.69, 137.24, 136.78, 128.56, 128.50, 128.33, 128.28, 128.06, 127.89, 127.78, 86.14, 81.47, 75.35, 73.41, 72.36, 72.28, 70.23, 63.11, 62.59, 60.74. DEPT-135 (CDCl_3_, 75 MHz): δ (ppm) positive 128.56, 128.50, 128.33, 128.28, 128.06, 127.89, 127.78, 86.14, 81.47, 75.35, 63.11, 62.59; negative 73.41, 72.36, 72.29, 70.23, 60.74. FTICR-MS *m/z*: 490.2230 [M+H]^+^ (C_29_H_32_NO_6_^+^ requires 490.2224).

## 4. Conclusions

In summary, the access to both C-6 epimers of protected L-homoDMDP was achieved with high efficiency. The (6*S*)-epimer *en-***7** was prepared in 44% overall yield from vinylpyrrolidine *en-***9** using the iodolactonization as a key step. The (6*R*)-epimer *en-***8** was synthesized in 59% overall yield from the known diol **13** through a 3-step-sequence-regioselective tritylation of diol **13**, lactonization to give oxazolidinone and acidic hydrolysis of the trityl ether. The stereochemistry confirmation of L-homoDMDP was achieved through 2D-NMR and X-ray data based on the bicyclic oxazolidinone intermediate *en*-**8**. This work will lead to the collective synthesis of homoDMDP-related natural products and their analogues, and potentially to the unambigous disclosure of their stereochemistry.

## Supplementary Materials

^1^H and ^13^C-NMR spectra of compounds *en-7* and *en-8 *were available free of charge. Supplementary materials can be accessed at: http://www.mdpi.com/1420-3049/18/6/6723/s1.
