# Supplementary material for: General Intermediates for the Synthesis of 6-C-Alkylated DMDP-Related Natural Products
Source: Molecules. 2013 Jun 6;18(6):6723–33. doi: 10.3390/molecules18066723 (PMC6269708; doi:10.3390/molecules18066723)
Supplement: Supplementary file 1 [file molecules-18-06723-s001.pdf]

Supplementary Materials

Figure S1. ^{1}H-NMR of oxazolidinone *en*-**7**.

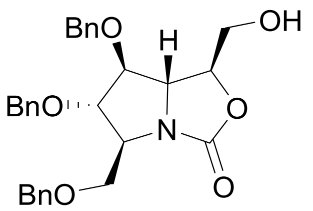

***en*-7**

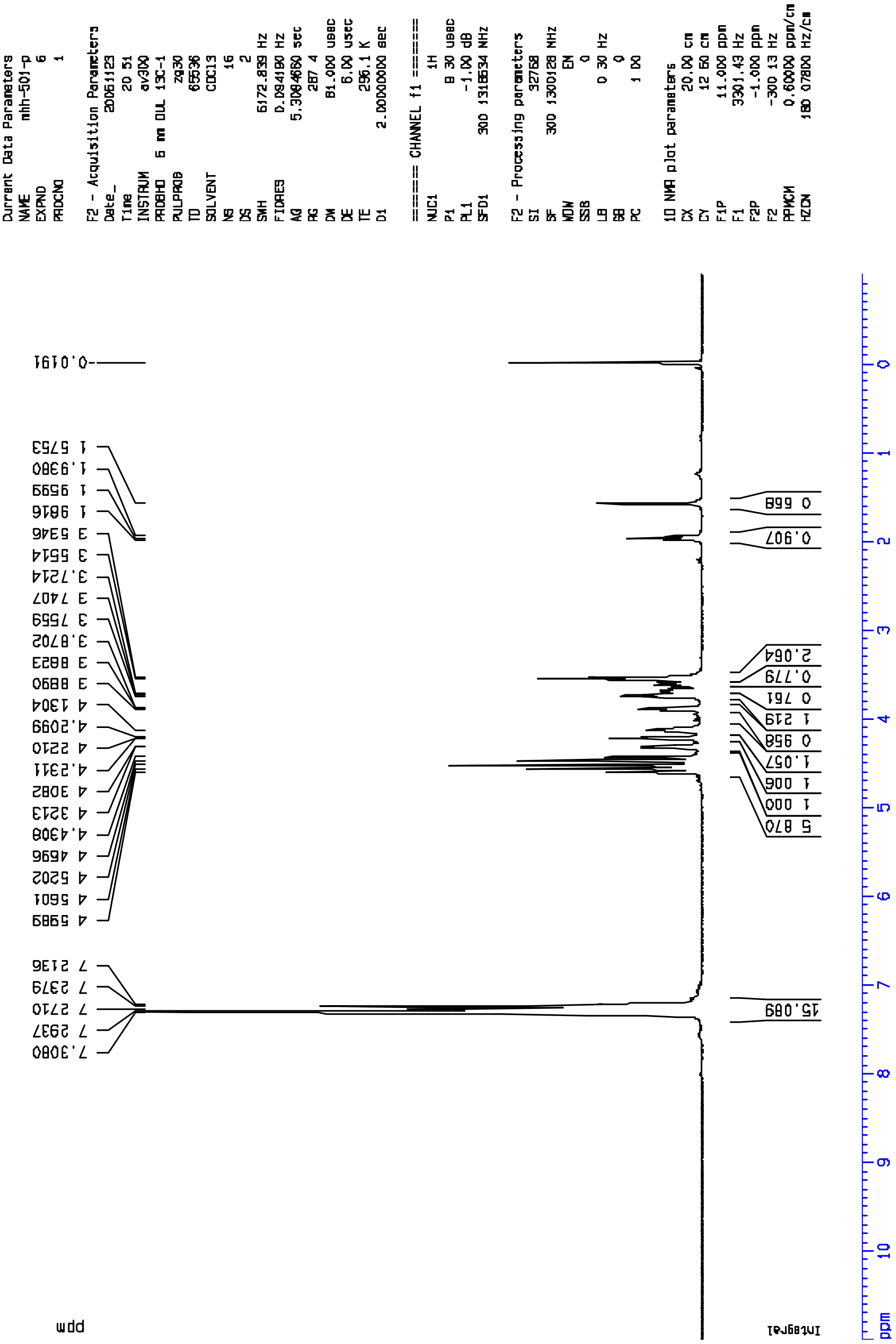

Figure S2. ^{13}C-NMR of oxazolidinone *en*-**7**.

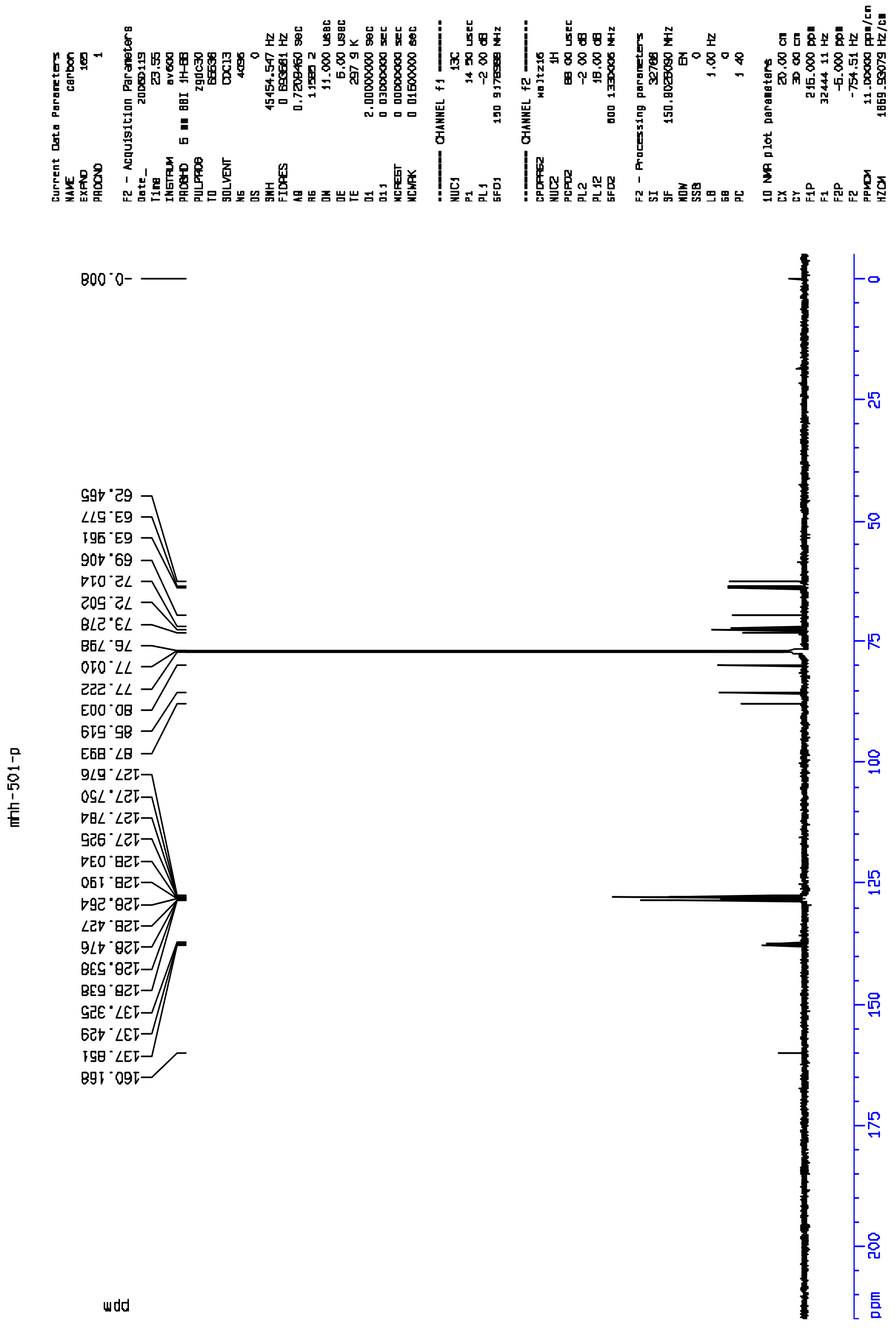

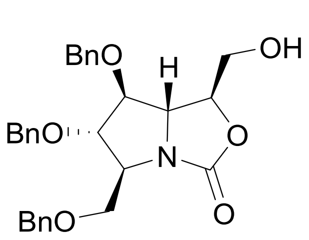

***en*-7**

Figure S3. ^{1}H-^{1}H COSY of oxazolidinone *en*-**7**.

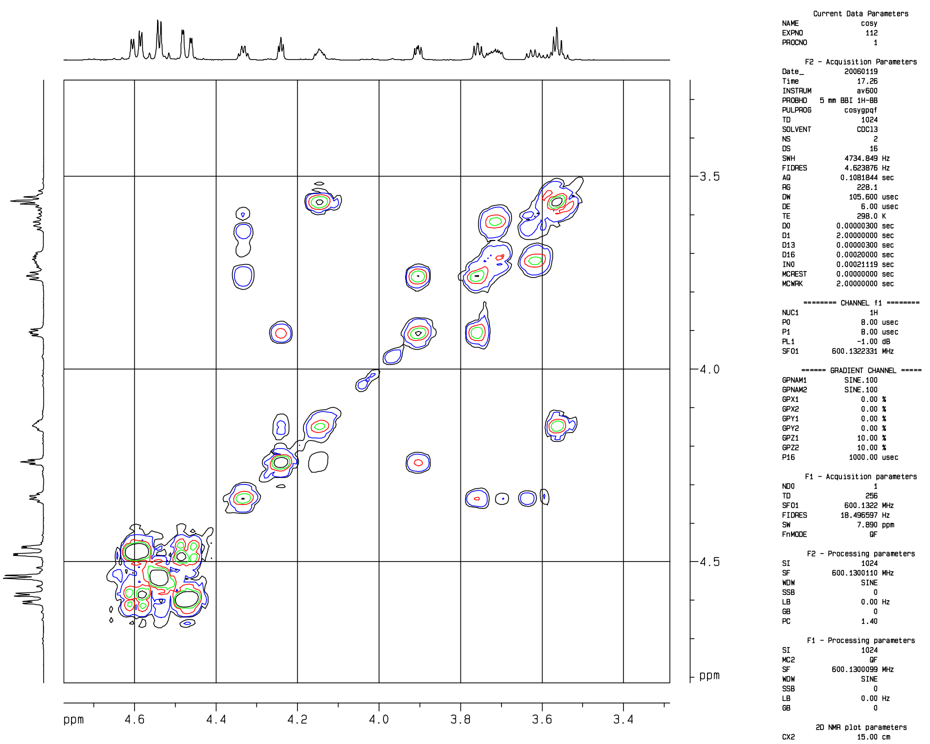

Figure S4. NOESY of oxazolidinone *en*-**7**.

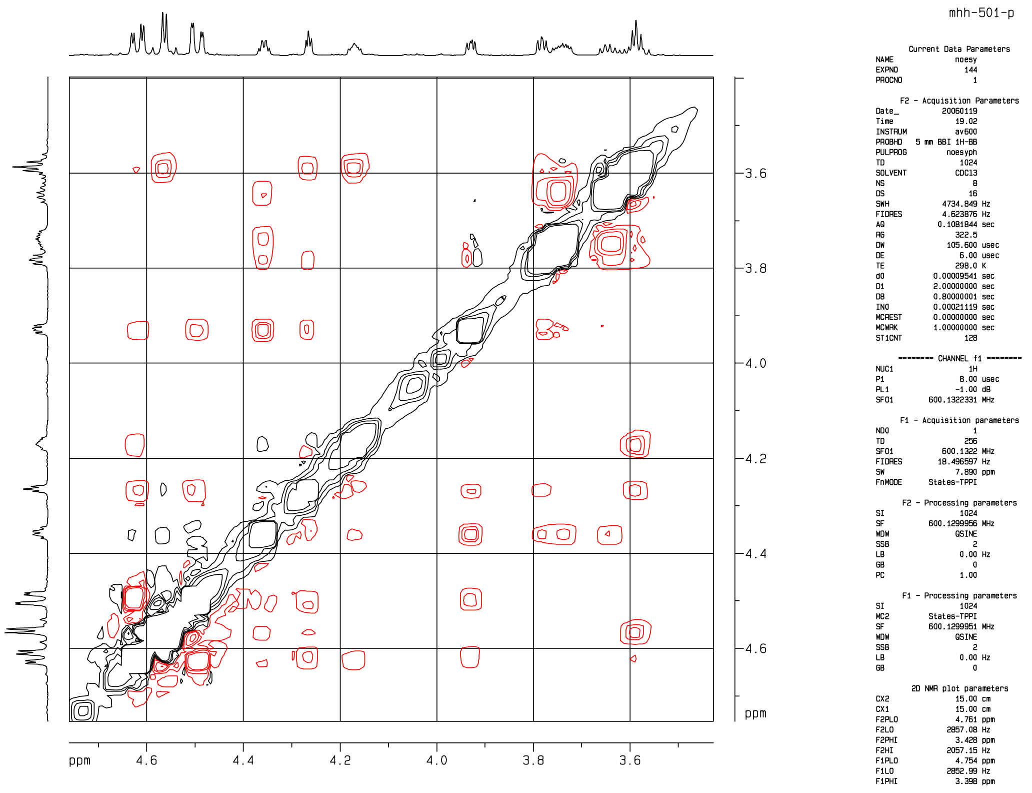

Figure S5. TOCSY of oxazolidinone *en*-**7**.

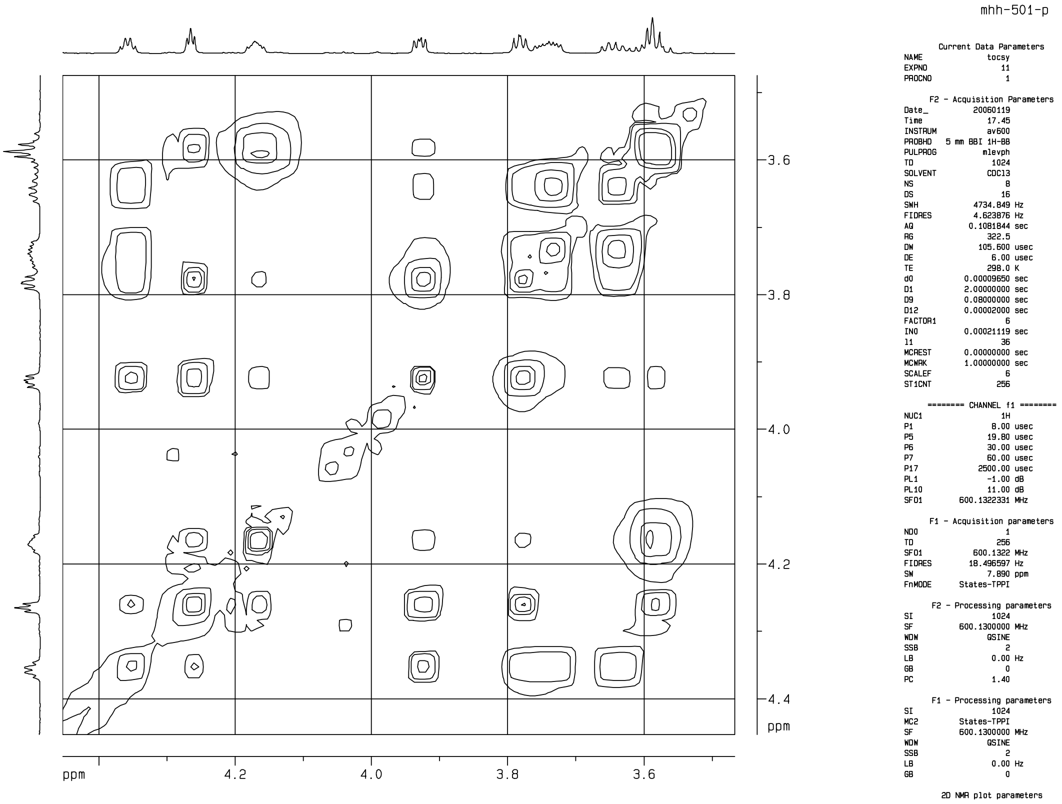

Figure S6. HMBC of oxazolidinone *en*-**7**.

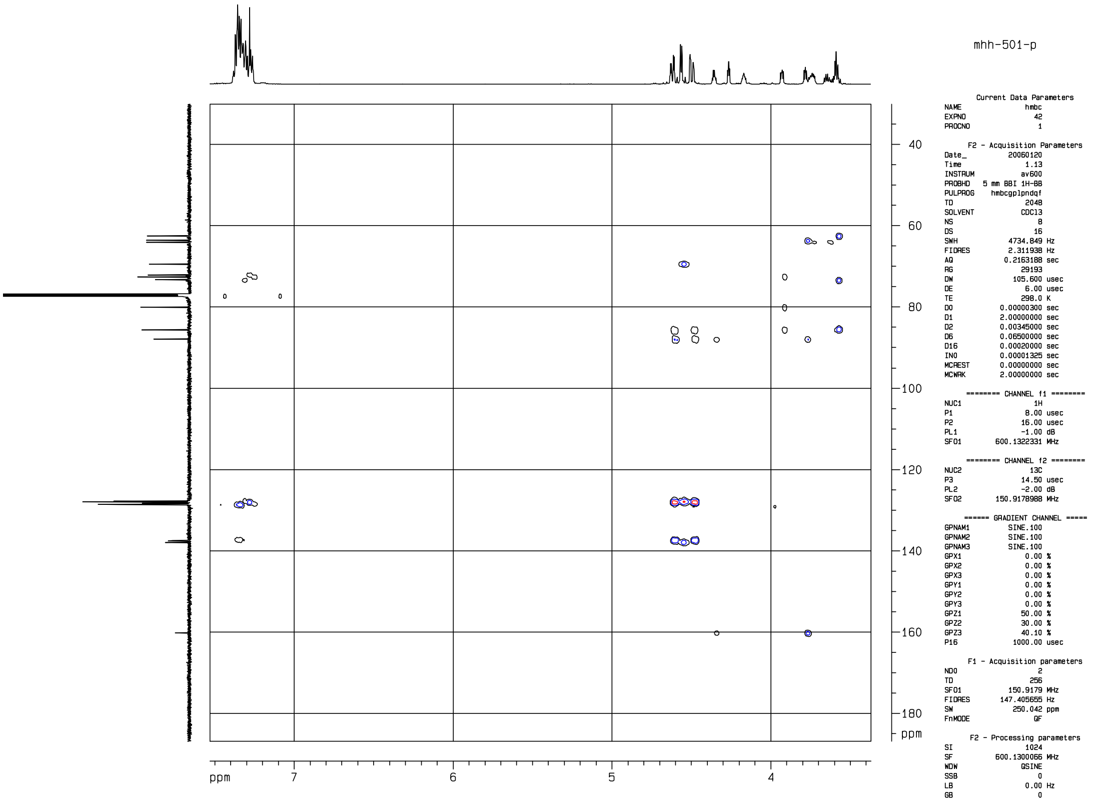

Figure S7. ^{1}H-NMR of oxazolidinone *en*-**8**.

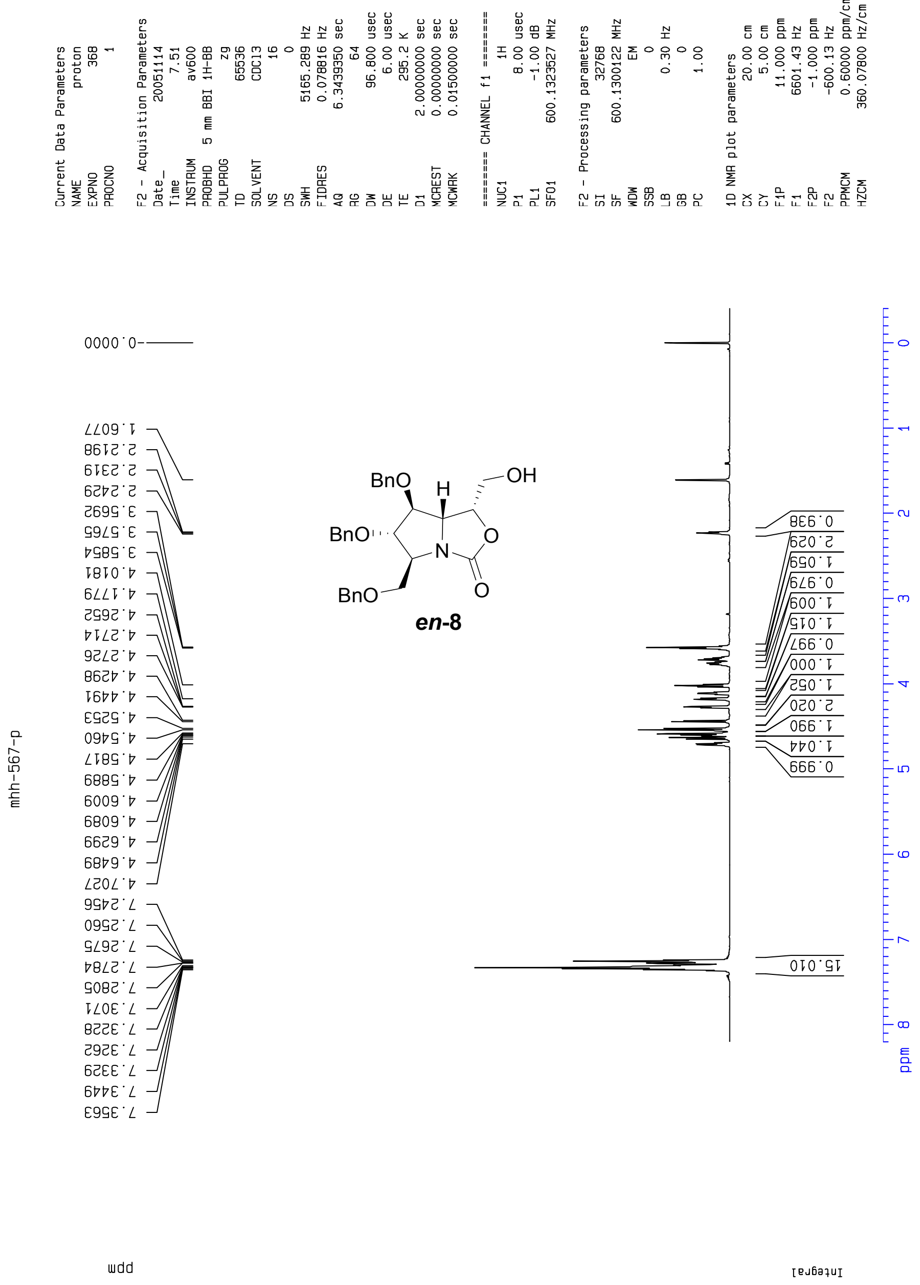

Figure S8. ^{13}C-NMR of oxazolidinone *en*-**8**.

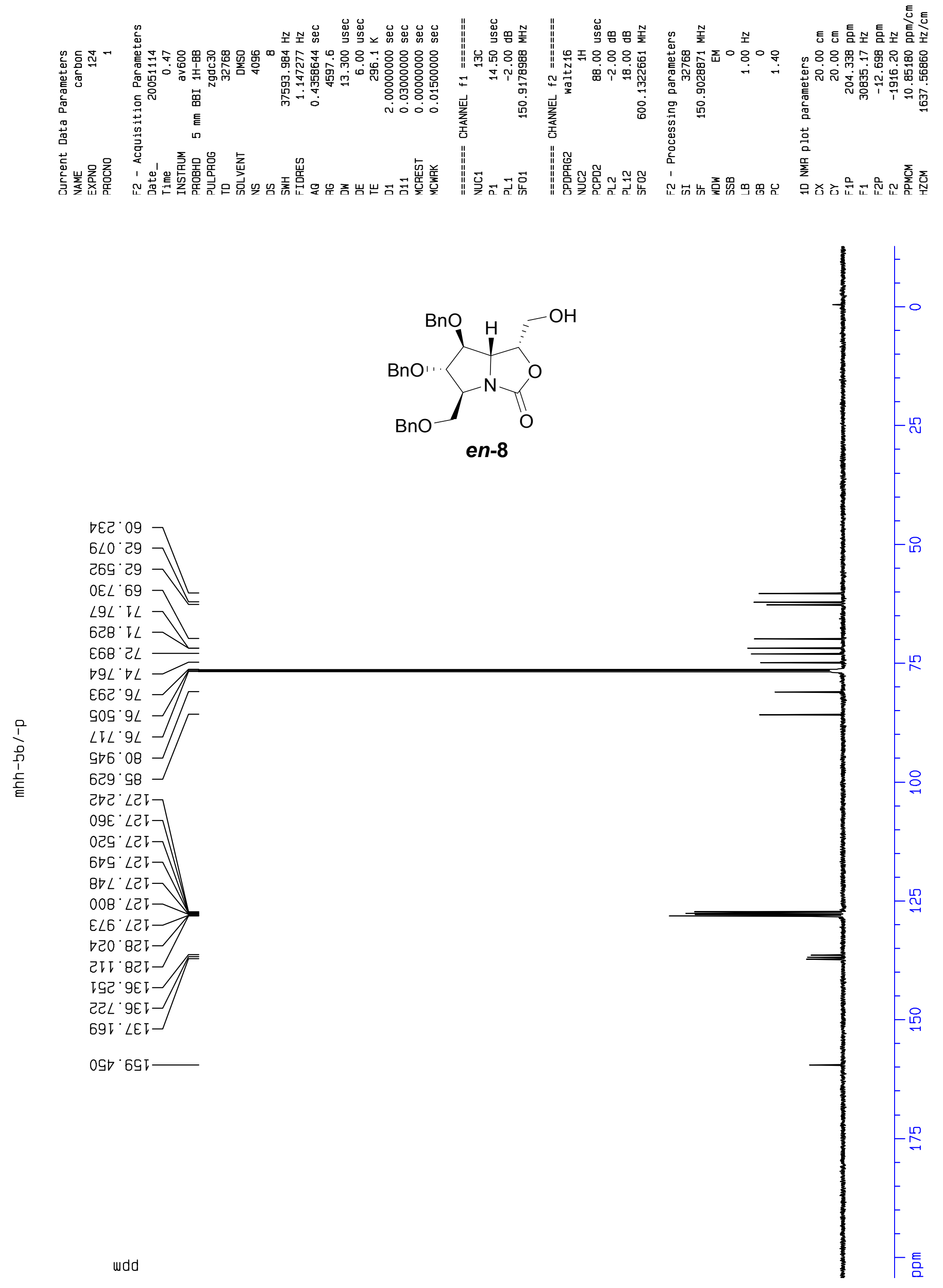

Figure S9. X-ray of oxazolidinone **11**.

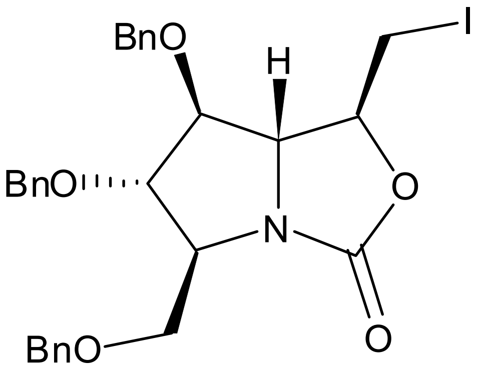

356

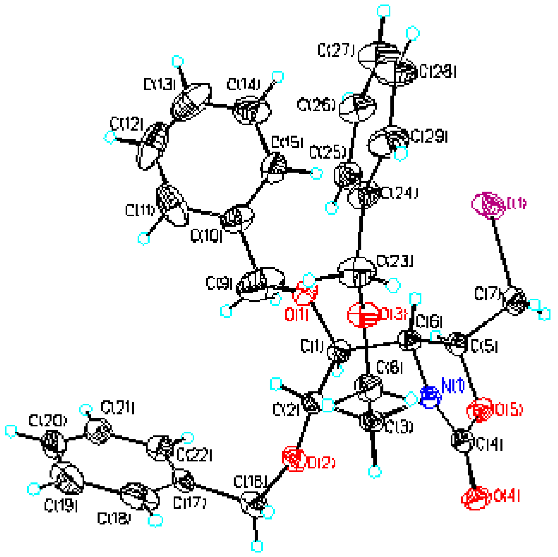

Table S1. Crystal data and structure refinement for **11**.

Identification code	a	
Empirical formula	$C_{29}H_{30}INO_5$	
Formula weight	599.44	
Temperature	293(2) K	
Wavelength	0.71073 Å	
Crystal system, space group	Orthrhombic, P2(1)2(1)2(1)	
	a = 9.3205(19) Å	= 90 °
Unit cell dimensions	*b* =10.511(2) Å	= 90 °
	c = 28.170(6) Å	= 90 °
Volume	2759.8(10) Å ^{3}	
Z, Calculated density	4, 1.443 Mg/m^3	
Absorption coefficient	1.198 mm^{-1}	
F(000)	1216	
Crystal size	0.76 × 0.25 × 0.23 mm	
Theta range for data collection	1.45 to 27.48 °	
Limiting indices	−12≤h≤12, −13≤k≤13, −35≤l≤36	
Reflections collected / unique	24775/3578 [R(int) = 0.0559]	
Completeness to theta = 27.48	100.0%	
Absorption correction	Empirical	
Max. and min. transmission	0.7677 and 0.4622	
Refinement method	Full-matrix least-squares on F^2	
Data/restraints/parameters	3578/0/326	
Goodness-of-fit on F^2	0.983	
Final R indices [$I > 2sigma(I)$]	R1 = 0.0373, wR2 = 0.0851	
R indices (all data)	R1 = 0.0689, wR2 = 0.1137	
Absolute structure parameter	0.47(5)	
Largest diff. peak and hole	0.413 and −0.300 e. $Å^{-3}$	

Table S2. Atomic coordinates (× 10^4) and equivalent isotropic displacement parameters ($Å^2 \times 10^3$) for **356** U(eq) is defined as one third of the trace of the orthogonalized Uij tensor.

	**X**	**Y**	**Z**	**U(eq)**
I(1)	974(1)	7666(1)	1103(1)	94(1)
O(1)	3747(5)	4303(4)	1336(2)	66(1)
O(2)	2956(5)	2211(5)	2238(1)	73(1)
O(3)	1053(5)	2564(3)	868(1)	67(1)
O(4)	930(6)	3271(4)	2471(2)	82(1)
O(5)	9(6)	5176(4)	2277(1)	68(1)
N(1)	275(5)	3576(4)	1759(2)	50(1)
C(1)	2789(7)	3990(6)	1706(2)	55(2)
C(2)	2548(6)	2546(6)	1766(2)	55(1)
C(3)	905(7)	2304(5)	1699(2)	54(1)
C(4)	279(8)	3920(6)	2193(2)	61(2)
C(5)	816(7)	5717(5)	1885(2)	55(1)
C(6)	1294(7)	4539(5)	1601(2)	52(2)
C(7)	169(8)	6613(6)	1621(2)	69(2)
C(8)	517(8)	1741(5)	1219(2)	68(2)
C(9)	5094(13)	4466(17)	1423(3)	169(6)
C(10)	5960(10)	5009(9)	1031(3)	90(2)
C(11)	7336(15)	4499(10)	906(5)	134(5)
C(12)	8118(15)	5016(17)	543(7)	151(7)
C(13)	7599(19)	5934(18)	307(6)	149(7)
C(14)	6317(15)	6421(12)	397(4)	126(4)
C(15)	5554(9)	6015(11)	761(3)	106(3)
C(16)	3152(10)	908(10)	2312(3)	110(3)
C(17)	4619(8)	458(8)	2126(3)	70(2)
C(18)	4709(12)	786(10)	1929(3)	103(3)
C(19)	6128(14)	1264(9)	1782(3)	109(3)
C(20)	7233(12)	421(10)	1838(3)	92(3)
C(21)	7070(10)	735(10)	2015(3)	91(3)
C(22)	5791(10)	1178(9)	2159(2)	82(2)
C(23)	834(10)	2133(6)	403(2)	86(2)
C(24)	1207(9)	3165(7)	61(2)	72(2)
C(25)	2194(9)	4086(7)	175(2)	73(2)
C(26)	2487(11)	5045(9)	140(3)	94(3)
C(27)	1880(13)	5084(9)	571(3)	109(3)
C(28)	916(13)	4160(10)	687(3)	109(3)
C(29)	567(10)	3198(9)	381(2)	98(3)

Table S3. Bond lengths [Å] and angles [°] for **356**.

I(1)-C(7)	2.118(7)	C(21)-C(22)	1.342(11)	C(1)-C(6)-C(5)	117.6(5)
O(1)- C(9)	1.290(11)	C(23)-C(24)	1.492(9)	C(5)-C(7)-I(1)	111.0(4)
O(1) -C(1)	1.412(7)	C(24)-C(25)	1.373(10)	O(3)-C(8)-C(3)	107.5(5)
O(2)- C(16)	1.397(11)	C(24)-C(29)	1.382(10)	O(1)-C(9)-C(10)	116.0(9)
O(2)-C(2)	1.427(6)	C(25)-C(26)	1.371(10)	C(15)-C(10)-C(11)	113.9(9)
O(3)-C(23)	1.401(7)	C(26)-C(27)	1.338(12)	C(15)-C(10)-C(9)	124.4(11)
O(3)-C(8)	1.405(7)	C(27)-C(28)	1.363(13)	C(11)-C(10)-C(9)	121.7(12)
O(4)-C(4)	1.202(7)	C(28)-C(29)	1.368(12)	C(12)-C(11)-C(10)	120.7(12)
O(5)-C(4)	1.364(8)	C(9)-O(1)-C(1)	120.5(7)	C(13)-C(12)-C(11)	119.4(16)
O(5)-C(5)	1.461(7)	C(16)-O(2)-C(2)	114.5(6)	C(12)-C(13)-C(14)	122.6(17)
N(1)-C(4)	1.375(7)	C(23)-O(3)-C(8)	113.9(5)	C(15)-C(14)-C(13)	120.6(13)
N(1)-C(6)	1.458(7)	C(4)-O(5)-C(5)	110.1(4)	C(14)-C(15)-C(10)	122.5(10)
N(1)-C(3)	1.471(7)	C(4)-N(1)-C(6)	109.5(5)	O(2)-C(16)-C(17)	111.5(8)
C(1)-C(6)	1.537(9)	C(4)-N(1)-C(3)	119.5(4)	C(22)-C(17)-C(18)	120.0(8)
C(1)-C(2)	1.543(8)	C(6)-N(1)-C(3)	109.6(4)	C(22)-C(17)-C(16)	122.1(8)
C(2)-C(3)	1.564(9)	O(1)-C(1)-C(6)	110.1(5)	C(18)-C(17)-C(16)	117.9(9)
C(3)-C(8)	1.518(8)	O(1)-C(1)-C(2)	113.7(5)	C(17)-C(18)-C(19)	118.4(8)
C(5)-C(7)	1.511(8)	C(6)-C(1)-C(2)	105.0(5)	C(20)-C(19)-C(18)	115.0(8)
C(5)-C(6)	1.541(7)	O(2)-C(2)-C(1)	107.8(5)	C(21)-C(20)-C(19)	123.5(9)
C(9)-C(10)	1.483(12)	O(2)-C(2)-C(3)	109.5(5)	C(20)-C(21)-C(22)	122.3(11)
C(10)-C(15)	1.355(13)	C(1)-C(2)-C(3)	106.8(5)	C(17)-C(22)-C(21)	120.7(9)
C(10)-C(11)	1.433(16)	N(1)-C(3)-C(8)	111.3(5)	O(3)-C(23)-C(24)	109.5(5)
C(11)-C(12)	1.369(18)	N(1)-C(3)-C(2)	103.2(5)	C(25)-C(24)-C(29)	118.8(7)
C(12)-C(13)	1.27(2)	C(8)-C(3)-C(2)	113.9(5)	C(25)-C(24)-C(23)	121.2(6)
C(13)-C(14)	1.324(19)	O(4)-C(4)-O(5)	121.9(6)	C(29)-C(24)-C(23)	120.0(7)
C(14)-C(15)	1.320(14)	O(4)-C(4)-N(1)	128.3(6)	C(26)-C(25)-C(24)	120.1(7)
C(16)-C(17)	1.539(12)	O(5)-C(4)-N(1)	109.8(5)	C(27)-C(26)-C(25)	121.6(9)
C(17)-C(22)	1.333(11)	O(5)-C(5)-C(7)	107.1(5)	C(26)-C(27)-C(28)	118.3(9)
C(17)-C(18)	1.423(13)	O(5)-C(5)-C(6)	103.4(4)	C(27)-C(28)-C(29)	122.1(8)
C(18)-C(19)	1.474(14)	C(7)-C(5)-C(6)	114.9(5)	C(28)-C(29)-C(24)	119.0(8)
C(19)-C(20)	1.368(14)	N(1)-C(6)-C(1)	105.7(4)		
C(20)-C(21)	1.323(13)	N(1)-C(6)-C(5)	102.1(4)		

Table S4. Anisotropic displacement parameters (Å^2 × 10^3) for **356**. The anisotropic displacement factor exponent takes the form: $-2\pi^2$ [$h^2 a^{*2}$ U11 + ... + 2 h k a* b* U12].

	U11	U22	U33	U23	U13	U12
I(1)	132(1)	54(1)	96(1)	17(1)	5(1)	1(1)
O(1)	54(3)	73(3)	71(2)	10(2)	11(2)	9(2)
O(2)	80(3)	86(3)	53(2)	6(2)	1(2)	26(3)
O(3)	95(3)	51(2)	54(2)	9(2)	4(2)	8(3)
O(4)	102(4)	68(3)	76(2)	17(2)	39(3)	13(3)
O(5)	87(3)	59(2)	58(2)	−6(2)	23(3)	8(3)
N(1)	59(3)	42(2)	47(2)	1(2)	7(2)	6(2)
C(1)	63(4)	55(3)	46(3)	−8(3)	5(3)	2(3)
C(2)	66(4)	55(3)	44(2)	2(3)	6(3)	13(3)
C(3)	69(4)	40(2)	52(3)	4(2)	10(3)	4(4)
C(4)	67(4)	52(3)	64(4)	6(3)	11(3)	14(3)
C(5)	65(4)	47(3)	53(3)	−6(2)	5(3)	10(3)
C(6)	69(4)	37(2)	50(3)	0(2)	7(3)	0(3)
C(7)	76(4)	46(3)	86(4)	−5(3)	5(4)	14(3)
C(8)	83(5)	42(3)	78(4)	4(3)	9(3)	1(3)
C(9)	117(8)	273(17)	116(7)	26(10)	−23(7)	−108(11)
C(10)	67(5)	118(6)	86(5)	2(5)	−1(5)	−42(6)
C(11)	117(10)	85(6)	201(12)	−7(7)	−79(10)	2(7)
C(12)	68(7)	172(15)	213(18)	−91(13)	39(10)	−31(10)
C(13)	118(14)	189(17)	140(11)	−41(11)	48(10)	−76(12)
C(14)	135(10)	114(8)	128(8)	32(7)	5(8)	−34(9)
C(15)	72(6)	141(8)	106(6)	9(7)	12(5)	17(6)
C(16)	88(6)	122(8)	121(7)	65(7)	9(5)	29(6)
C(17)	58(4)	78(5)	74(4)	32(4)	−3(3)	18(4)
C(18)	105(7)	99(7)	104(6)	44(6)	−40(6)	−36(6)
C(19)	148(9)	79(5)	100(6)	7(5)	−12(7)	20(8)
C(20)	114(7)	93(6)	69(5)	10(5)	19(5)	50(7)
C(21)	73(5)	129(8)	70(4)	33(5)	−2(4)	23(6)
C(22)	83(6)	94(5)	68(4)	14(4)	1(4)	8(6)
C(23)	133(7)	65(4)	59(3)	−18(3)	8(5)	−16(5)
C(24)	94(5)	68(4)	53(3)	−15(3)	16(4)	−2(5)
C(25)	86(5)	72(4)	60(3)	−4(3)	6(4)	3(4)
C(26)	117(7)	91(5)	74(5)	−4(4)	13(5)	−24(6)
C(27)	162(9)	95(6)	69(5)	1(5)	6(6)	−26(8)
C(28)	152(8)	127(7)	49(4)	0(4)	−13(6)	−13(9)
C(29)	131(8)	110(6)	54(4)	−18(4)	10(4)	−26(6)

Table S5. Hydrogen coordinates (× 10^4) and isotropic displacement parameters (Å^2 × 10^3) for **356**.

	x	y	z	U(eq)
H(1A)	3146	4345	2005	65
H(2A)	3108	2065	1532	66
H(3A)	558	1741	1951	65
H(5A)	1654	6178	2006	66
H(6A)	1184	4693	1259	62
H(7A)	−614	7194	1844	83
H(7B)	−924	6129	1468	83
H(8A)	939	902	1185	81
H(8B)	−516	1662	1190	81
H(9A)	5504	3650	1510	202
H(9B)	5183	5022	1696	202
H(11A)	7701	3810	1074	161
H(12A)	9021	4695	469	181
H(13A)	8139	6279	61	179
H(14A)	5951	7056	201	151
H(15A)	4706	6435	836	128
H(16A)	2396	442	2151	132
H(16B)	3083	726	2648	132
H(18A)	3892	−1286	1893	123
H(19A)	6261	−2078	1660	130
H(20A)	8148	−675	1747	111
H(21A)	7870	1260	2040	109
H(22A)	5723	1996	2282	98
H(23A)	−162	1888	362	103
H(23B)	1429	1393	344	103
H(25A)	2664	4058	466	87
H(26A)	3123	5686	−53	113
H(27A)	2111	5724	−785	130
H(28A)	481	4184	−984	131
H(29A)	−90	2575	−469	118
